# Glucanases and Chitinases in *Mangifera indica*: Identification, Classification, Phylogeny, and Expression Analysis of Defense Genes against *Colletotrichum* spp.

**DOI:** 10.3390/molecules29153556

**Published:** 2024-07-28

**Authors:** María Isabel Jiménez-Maldonado, María Auxiliadora Islas-Osuna, Josefina León-Félix, Juan Manuel Tovar-Pedraza, María Dolores Muy-Rangel

**Affiliations:** 1 Centro de Investigación en Alimentación y Desarrollo, Coordinación Culiacán, Carretera a El Dorado km 5.5, Campo El Diez, Culiacán CP 80110, Sinaloa, Mexico; isabel.jimenez@estudiantes.ciad.mx (M.I.J.-M.); ljosefina@ciad.mx (J.L.-F.); juan.tovar@ciad.mx (J.M.T.-P.); 2 Centro de Investigación en Alimentación y Desarrollo, Coordinación de Tecnología de Alimentos de Origen Vegetal, Carretera Gustavo Enrique Astiazarán Rosas, No. 46, La Victoria, Hermosillo CP 83304, Sonora, Mexico; islasosu@ciad.mx

**Keywords:** *Colletotrichum asianum*, *Colletotrichum siamense*, chitinases, glucanases, mango, Ataulfo, genes

## Abstract

Plant glucanases and chitinases are defense proteins that participate in pathogenesis; however, very little is known about the glucanase (*GLUC*) and chitinase (*CHIT*) gene families in mango. Some mango cultivars are of great economic importance and can be affected by anthracnose, a postharvest disease caused by fungi of the genus *Colletotrichum* spp. This study identified and characterized 23 putative glucanases and 16 chitinases in the mango genome cv. Tommy Atkins. We used phylogenetic analyses to classify the glucanases into three subclasses (A, B, and C) and the chitinases into four classes (I, II, IV, and V). Information on the salicylic, jasmonic acid, and ethylene pathways was obtained by analyzing the *cis*-elements of the *GLUC* and *CHIT* class I and IV gene promoters. The expression profile of *GLUC*, *CHIT* class I, and *CHIT* class IV genes in mango cv. Ataulfo inoculated with two *Colletotrichum* spp. revealed different profile expression related to these fungi’s level of virulence. In general, this study provides the basis for the functional validation of these target genes with which the regulatory mechanisms used by glucanases and chitinases as defense proteins in mango can be elucidated.

## 1. Introduction

Mango (*Mangifera indica* L.) belongs to the Anacardiaceae family and is the most predominant tropical fruit produced worldwide [1,2]. However, the quality of mango pre- and postharvest can be affected by diseases, such as anthracnose, which is caused by at least seven cryptic species of the *Colletotrichum gloeosporioides* complex (*C. siamense*, *C. asianum*, *C. tropicale*, *C. alienum*, *C. fructicola*, *C. chrysophilum*, and *C. queenslandicum*) in Mexico [1,3,4]. *C. asianum* and *C. siamense* have the highest pathogenicity among mango cultivars. They have been reported in different parts of the world and classified as having high virulence [1,2,3,4,5,6,7,8,9]. In places with high relative humidity, the incidence of this disease can reach up to 100% in fruits [1,4].

Anthracnose affects terminal branches, inflorescences, leaves, and growing fruits [4,9]. This fungus can occur in latent form when the fruits are small, resuming its infection once physiological maturity is reached [10,11]. The infective cycle begins with establishing the conidium on the fruit’s surface; subsequently, the melanized appressorium penetrating the cuticle is formed, producing a latent subcuticular hypha. This hypha develops until the fruit matures and causes black, irregular, sunken lesions or spots that can cover a large part of the fruit [4,10]. Consequently, during postharvest storage, poor-quality fruits are generated, leading to high losses in the production and marketing of mango [2,10]. The varied responses of mango cultivars to this disease in different parts of the world have prevented plant resistance to this fungus and reduced the effectiveness of antifungal treatments [9]. This makes it necessary to search for effective alternatives to combat these phytopathogens by understanding the signaling and plant defense mechanisms. The differences generated during the development of symptoms in mango cultivars are associated with the relationship between plant immunity and pathogen recognition.

During the plant–pathogen interaction, the plant cell has two interaction mechanisms: pathogen-associated molecular pattern (PAMP)-triggered immunity (PTI) and effector-triggered immunity (ETI) [12]. PTI uses receptors that recognize PAMPs, and ETI recognizes effectors through resistance proteins [12,13]. Hormonal pathways are also activated by regulating and changing the concentration or sensitivity of salicylic acid (SA), jasmonic acid (JA), and ethylene (ET) [14]. These generate high expression in genes encoding defense proteins, such as those related to pathogenesis (PRs) [15]. PRs, such as glucanases (PR2) and chitinases (PR3, PR4, PR8, PR11), prevent infection and limit the activity of pathogenic agents [16,17,18].

The classification of β-glucanases and chitinases is based on their sequence of amino acids and common catalytic domains, as well as their distribution within the glycoside hydrolase (GH) families according to the carbohydrate-active enzymes (CAZy) database [19]. Depending on the type of glycoside bond they hydrolyze, there are β-1,4-glucanases, β-1,3-glucanases, and β-1,3-1,4-glucanases [20]. β-1,4-glucanases are divided into three subclasses (A, B, and C) of the GH9 family [20]. β-1,3-glucanases are classified into five groups (I, II, III, IV, and V) that are distinguished by being from the GH17 family [20]. Finally, β-1,3-1,4-glucanases have varied sequences and are shaped similar to the group V of the β-1,3-glucanases; they are also from the GH17 family [20]. Chitinases are classified by the similarity of the amino acid sequences and their catalytic domains, which is why they are divided into five classes (I–V) [18]. Classes III and V belong to the GH18 family, and classes I, II, and IV belong to the GH19 family [18].

Fungal cell walls are primarily made up of β-glucans and chitin, which are degraded by enzymes, such as β-glucanases and plant chitinases, as a defense mechanism against the pathogen [17,21,22]. To date, no studies have reported mango β-glucanases and chitinases involved in the response to the biotic stress caused by *Colletotrichum* spp.; only the expression of genes that respond to biotic stress stimulation has been analyzed. In mango fruits infected with *C. gloeosporioides*, the resistance induced by β-aminobutyric acid has been analyzed, and an increase in the activity of chitinase and glucanase enzymes has been demonstrated 2 days post-induction. Additionally, based on quantitative proteomics, 181 proteins differentially regulated by the fungus have been obtained, of which 40 increased in response to *C. gloeosporioides* [23]. Hong et al. [24] conducted a transcriptomic analysis in which they analyzed the peel of mango cv. Zill infected with *C. gloeosporioides*. One of their important findings was the overexpression of three genes encoding possible PRs such as protein FATTY ACID EXPORT 1, chloroplastic-like (comp22382_c0_seq1; GenBank: GBCV01028555), a mannan endo-1,4-beta-mannosidase 2-like (comp23077_c0_seq1; GenBank: GBCV01029236); and comp24098_c0_seq1 (GenBank: not found). However, these PR genes do not include glucanases or chitinases.

Although several mango genomes are available, we chose to work with the Tommy Atkins genome (https://mangobase.org, accessed on 4 January 2023), which has a helpful platform with annotations. The objectives of this study were to identify and characterize *M. indica* glucanases and chitinases, analyze phylogenetic relationships with orthologous proteins induced by fungi, and analyze the *cis*-elements of the promoter regions of these genes. In addition, we aimed to evaluate the gene expression profile of one glucanase and two chitinases in mango cv. Ataulfo infected with anthracnose (*C. siamense* and *C. asianum*) and to elucidate gene products that are part of the defense mechanism of the mango against *Colletotrichum* spp.

## 2. Results and Discussion

### 2.1. Classification and Characterization of Glucanases from M. indica

**Figure 1:**
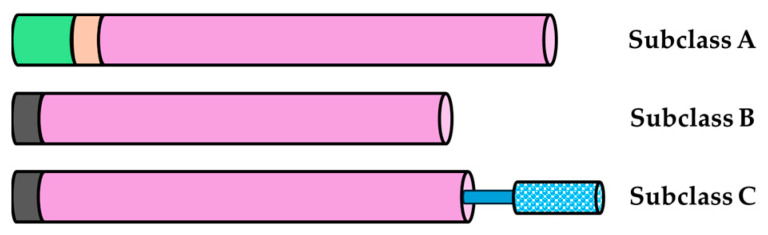
Schematic representation of the structure of β-1,4-glucanases of the GH9 family of plants. The subclasses are composed of the cytosolic domain (green), transmembrane domain (pink), signal sequence (dark gray), GH9 catalytic domain (purple), linker region (thick blue line), and CBM49 carbohydrate-binding module (blue dots) (adapted from Urbanowicz et al. [27]).

**Figure 2:**
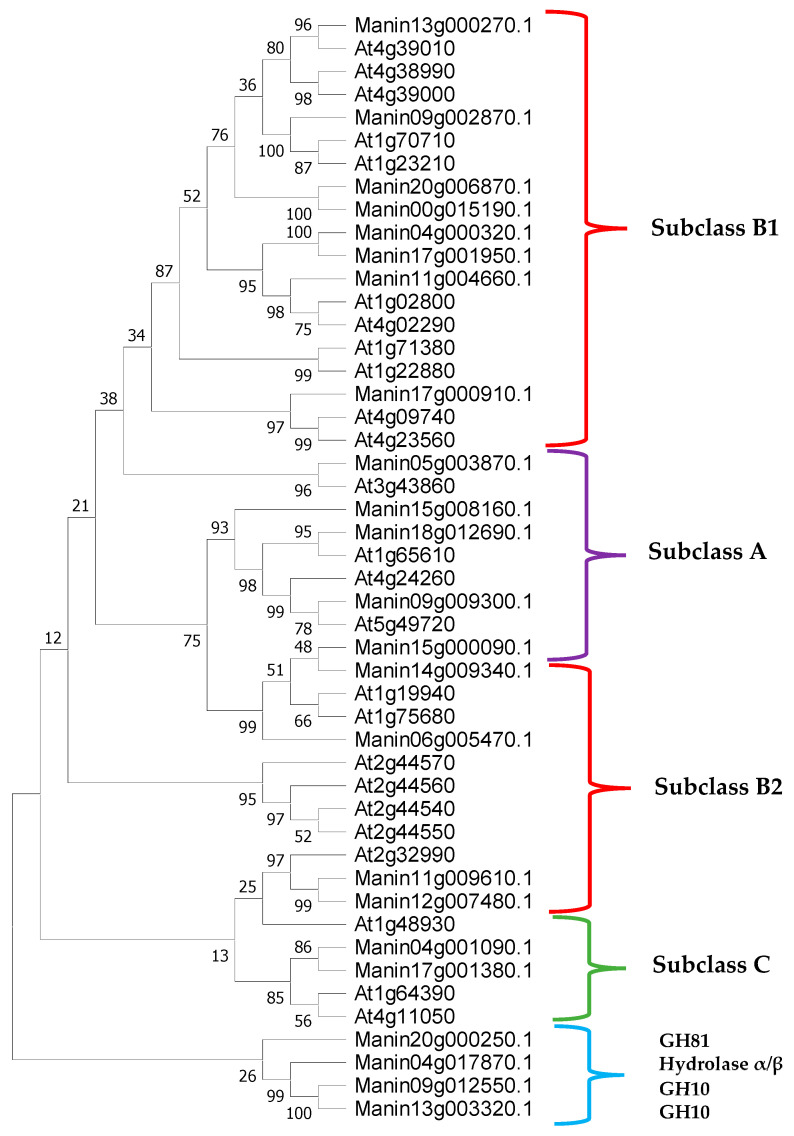
Phylogenetic tree of *M. indica* glucanases identified from the glycosylhydrolase family are GH9 (red/purple/green), GH10, GH81, and α/β hydrolase (blue). The subclasses of β-1,4-glucanases are represented as follows: purple = subclass A, red = subclass B, green = subclass C, and blue = glucanases from other families. The phylogenetic tree shows that the *M. indica* glucanases belong to *A. thaliana* subclasses. The GH9 family comprises subclasses A, B1, B2, and C, whereas the other GH families are grouped in the smaller clade. The percentage of trees in which the associated taxa clustered is shown next to the branches.

A total of 23 putative glucanases were identified from the genome of mango cv. Tommy Atkins [25], and by locating and predicting the conserved domains and sites, 19 were identified as β-1,4-glucanases. Through alignments and phylogenetic analyses, these were classified into subclasses according to the criteria described above for β-1,4-glucanases of the GH9 family of *Arabidopsis* (the model plant for the standardized nomenclature of β-1,4-glucanases) (Figure 1) [26,27]. Until now, no β-1,3-1,4-glucanases have been identified in *Arabidopsis*. In total, 5 β-1,4-glucanases of subclass A, 12 of subclass B (8 of class B1 and 4 of class B2), and 2 of subclass C were found in *M. indica* (Figure 2 and Table 1) [20,26,27,28]. Of the 23 glucanases, 4 presented different catalytic domains; there were 2 from the GH10 family, 1 from the GH81 family, and 1 from the α/β hydrolase. The length of the *M. indica* glucanases ranged from 426 to 1095 amino acids, of which 22 varied in size from 426 to 686 amino acids, and 4 contained more than 891 amino acids (Manin18g012690, Manin20g000250, Manin20g006870, and Manin13g003320). These long-sequence glucanases were observed to contain repeated catalytic domains.

We identified two glucanases with GH10 family domains that showed xylanase activity. Only two glucanases could hydrolyze endo-β-1,3-1,4 bonds: Manin04g017870, which contained two catalytic domains with endo-α/β hydrolase activity, and Manin20g000250, which had two endo-β-glucanase catalytic domains and two GH81 family domains at its N and C termini. The GH81 family, which has endo-β-1,3-glucanase activity, has a defense function against pathogens [19,20]. Cytosolic and transmembrane domains are contained only by the β-1,4-glucanases of subclass A, except for Manin05g003870, which, instead, presented a signal peptide. The carbohydrate-binding module CBM49 is only contained by the β-1,4-glucanases of subclass C. Meanwhile, the signal peptide sequence was predicted in some glucanases, such as Manin20g006870 and Manin13g003320; however, not all subclass B β-1,4-glucanases have signal peptides at their amino terminus.

### 2.2. Classification and Characterization of Chitinases from M. indica

Within the classification of chitinases, classes III and V contain domains from the GH18 family and are present in plants, animals, fungi, and viruses. In contrast, classes I, II, and IV contain domains from the GH19 family that are present only in plants [18,29,30]. Classes I and IV have a conserved chitin-binding domain (CBD) and a GH19 catalytic domain with an N-terminal region rich in cysteine and a C-terminal extension. Class IV chitinases and their sequence do not contain the latter extension; they are found with several deletions. Class II does not have an N-terminal region and lacks a CBD, but it is similar to the amino acid sequence of class I. Class III and V chitinases do not have a CBD and contain a GH18 catalytic domain; those of class V are most similar to fungal and bacterial chitinases and have a C-terminal extension [18,30,31].

**Figure 3:**
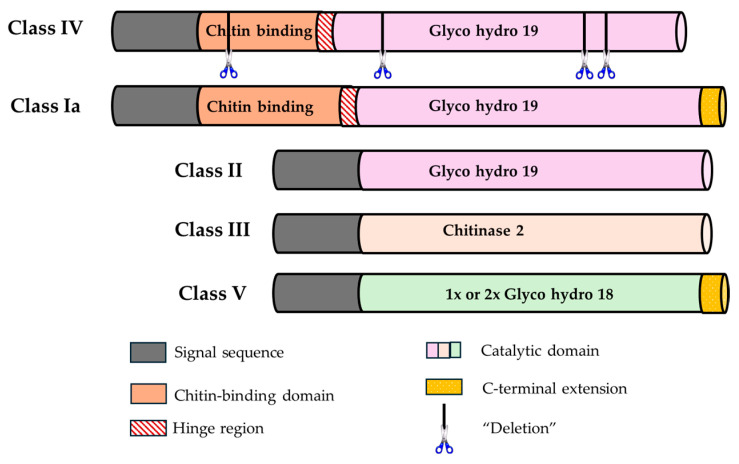
Schematic representation of the structural domains of plant chitinases (adapted from Passarinho and de Vries [30]).

**Figure 4:**
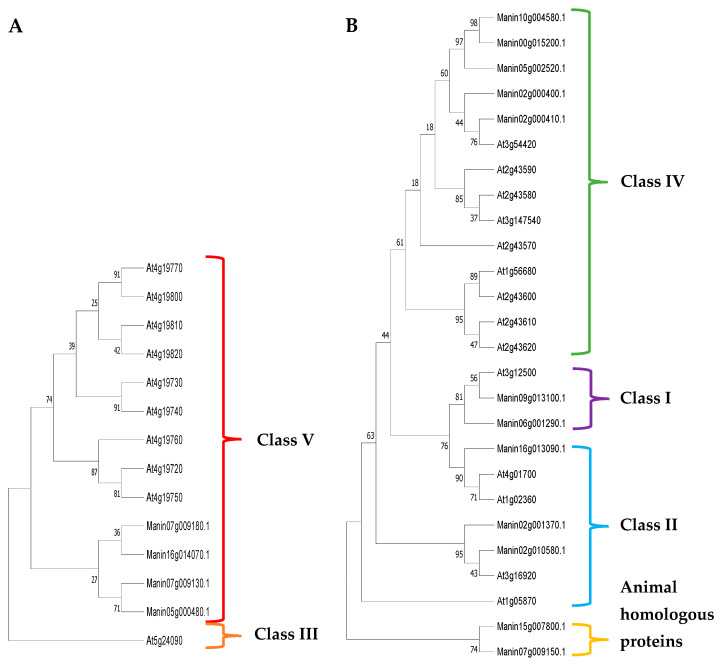
Phylogenetic trees of *M. indica* chitinases identified from the glycosylhydrolase family GH18 (**A**): class III (orange) and class V (red); and GH19 (**B**): class I (purple), class II (blue), class IV (green), and homologous animal proteins (yellow). The *M. indica* chitinases (Manin) were found in all but class III chitinases from *A. thaliana*. The percentage of trees in which the associated taxa clustered is shown next to the branches.

Sixteen putative chitinase sequences were obtained from the genome of mango cv. Tommy Atkins [25]. The GH18 and GH19 subfamilies shared little similarity in their amino acid sequences; thus, their alignment and phylogenetic analyses were performed separately. Four were classified as belonging to the GH18 subfamily and ten as belonging to the GH19 subfamily, according to the classification criteria for plant chitinases proposed by Passarinho and de Vries [30] and Cao et al. [18] in *Arabidopsis* (Figure 3). Therefore, two class I, three class II, five class IV, and four class V chitinases were identified in *M. indica*; however, two chitinases showed homology with animal proteins (Figure 4 and Table 2).

The length of the *M. indica* chitinases varied from 129 to 934 amino acids; the longest 5 contained more than 718 amino acids: Manin10g004580, Manin02g000400, Manin06g001290, Manin16g014070, and Manin07g009180. All class I, II, and IV chitinases were predicted to have a lysozyme function, and three (Manin05g000480, Manin07g009130.1, and Manin07g009180) of the four class V chitinases did not have this function. Two of these chitinases did not contain signal peptides (Manin05g000480 and Manin07g009130), while Manin07g009180 and Manin16g014070 of class V showed this signal sequence. Furthermore, all class I and IV chitinases presented CBDs.

### 2.3. Glucanases and Chitinases from M. indica with Possible Defense Function against Anthracnose

On average, β-glucan is the main component of fungal cell walls, making up 50–60% of its dry weight, while chitin accounts for between 10 and 20% of the dry weight [22,32,33]. Within the structure of the fungal wall, β-glucans are mainly formed in β-1,3 bonds (65–90%) and, to a lesser extent, in β-1,6 and β-1,3-1,4 bonds [22,32,33]. However, chitin is made up of N-acetyl-D-glucosamine (GlcNAc) with β-1,4 bonds [32,33].

It has been reported that endo-β-1,3-1,4-glucanases degrade Mixed Linked Glucan (MLG), which is present in monocotyledonous plants and cell walls of some fungi and bacteria [20,34,35,36,37,38,39]. However, the characterization of a few β-glucanases in dicotyledons has given us limited information about the signaling function that molecules generated from the hydrolysis of pathogens’ MLG could have [20]. Therefore, it has been suggested that the mixed-linked oligosaccharides of β-1,3-1,4-glucans derived from phytopathogens in dicotyledonous plants, which do not contain MLG, can act as PAMPs [20,35,38].

A previous study indicated that class I and IV chitinases are characterized by the presence of a conserved CBD that recognizes and hydrolyzes the endo-β-1,4 bonds of the GlcNAc subunits of chitin polymers [32]. In tomatoes, Jashni et al. [17] demonstrated that inoculation with *Cladosporium fulvum* and *Fusarium oxysporum* induced and positively regulated the expression of chitinase genes with CBD, i.e., class I and IV, and that the elimination of this catalytic domain significantly reduced their chitinase and antifungal activity. In addition, PAMPs, which are effector molecules that activate the plant immune system, are released from the hydrolysis of the fungal cell wall [33,40]. Other authors have reported that the overexpression of plant chitinases can reduce the symptoms of fungal diseases [41,42].

There is little information about mango glucanases and chitinases, and, thus far, there are no specific gene expression studies on mango PRs induced by species of the *C. gloeosporioides* complex. Hong et al. [24] reported the overexpression of two mango peel genes from cv. Zill infected with *C. gloeosporioides* encoding possible PRs other than glucanases and chitinases (GenBank: GBCV01028555, GBCV01029236). These nucleotide sequences were mapped onto the genome of mango cv. Tommy Atkins, but they were unrelated to glucanases and chitinases PRs (). These genes encode a protein FATTY ACID EXPORT 1, chloroplastic-like, and a mannan endo-1,4-beta-mannosidase 2-like.

Due to the lack of molecular information on mango, we used orthologous sequences of glucanases and chitinases reported in other plant organisms that were induced by biotic stress caused specifically by fungi. For β-1,3-glucanases, we used sequences from various dicotyledonous and monocotyledonous plant species, including *Arabidopsis*, jujube, potato, wheat, oats, rice, and barley (Table 3). The chitinases that responded to fungal infection were class I and IV in *Arabidopsis*, blackberry, and white pine. In addition, some class I chitinase genes were overexpressed in tea, lychee, and carrot, decreasing the damage caused by some fungal diseases (Table 3). We partially infer that mango’s endo-β-1,3-1,4-glucanases and class I and IV chitinases hydrolyze β-glucans [20] and chitin [17,32,33], respectively, which are both components of the fungal cell wall.

Only two glucanases that hydrolyze endo-β-1,3-1,4 bonds and two class I and five class IV chitinases were identified. Two phylogenetic analyses were carried out on these mango proteins—one with the two endo-β-1,3-1,4-glucanases and another with the seven chitinases—using their respective orthologous sequences from Table 3 (from the phylogenetic analyses). The glucanase Manin20g000250, the class I chitinase Manin09g013100, and class IV chitinase Manin05g002520 were chosen based on their greatest phylogenetic closeness with these orthologous glucanase and chitinase. Moreover, their relative expression was evaluated in mango cv. Ataulfo inoculated with *Colletotrichum* spp.

### 2.4. Prediction of cis Elements Acting in the Promoter Region of Endo-β-1,3-1,4-Glucanase and Class I and IV Chitinases Coding Genes from M. indica

1500 bp upstream of the ATG from the coding sequence (promoter regions) were chosen to identify *cis*-elements important for gene expression regulation of the three genes of interest. Those *cis* elements are responsible for interaction with transcription factors related to the defense response to biotic stress and the hormonal response. These are related to the presence of elicitor response elements in *Colletotrichum* spp. [51]. The response of hormones such as ethylene, salicylic, and jasmonic acid defines the pathways by which some pathogens, based on their lifestyle, carry out signal transduction during disease development [8,51]. Response factors to salicylic acid are related to immunity against biotrophic and hemibiotrophic pathogens, and response factors to ethylene and jasmonic acid are related to defense against necrotrophic pathogens [8].

**Figure 5:**
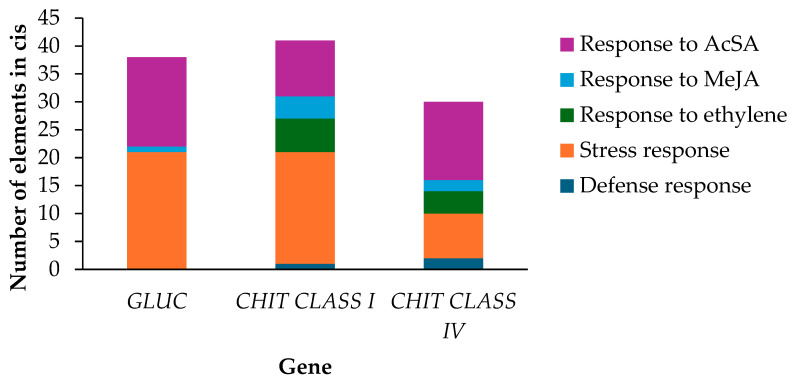
Frequency of appearance of *cis* elements in three sequences that correspond to the genes *GLUC*, *CHIT* class I, and *CHIT* class IV of *M. indica*. The vertical axis indicates the number of cis-regulatory elements, and the horizontal axis shows the genes.

Concerning the signaling pathway, transcription factors target the *cis* elements of the promoter regions to regulate gene expression at the transcriptional levels. In this study, *cis* elements were found in the three genes, glucanase (*GLUC*) and chitinase (*CHIT*) class I and IV of *M. indica* that respond to jasmonic acid and salicylic acid, as well as in response to stress (Figure 5). However, compared with the chitinase genes, the *GLUC* gene did not contain elements in response to ethylene or those in response to plant defense. Furthermore, the *GLUC* gene had the highest number of *cis* elements in response to salicylic acid (Figure 5), followed by the *CHIT* class IV gene. Response elements to ethylene and salicylic acid were found in the *CHIT* genes and marked by different signaling pathways, indicating that they can act on the lifestyle of hemibiotrophic *Colletotrichum* spp.

### 2.5. Gene Expression Profile of GLUC and CHIT class I and IV in Mango Fruits cv. Ataulfo in Response to C. siamense and C. asianum

**Figure 6:**
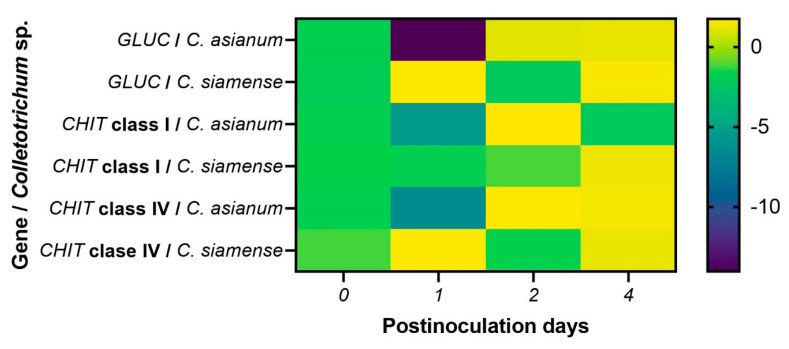
Expression profile of the glucanase gene (*GLUC* I) and chitinases class I (*CHIT* class I) and IV (*CHIT* class IV) of *M. indica* under biotic stress induced by *C. asianum* and *C. siamense*. Uninoculated mango fruits were used as internal control.

Relative gene expression analysis showed that the *GLUC* gene was expressed on day 1 in fruit inoculated with *C. siamense*, while it was expressed 2 days post-inoculation (dpi) in those inoculated with *C. asianum*. In addition, the response to both pathogens was maintained at 4 dpi. We observed an early induction in the expression of the *CHIT* class I gene in fruits inoculated with *C. asianum* compared to those inoculated with *C. siamense*, in which expression occurred at 4 dpi. The *CHIT* class IV gene was expressed early on day 1 in fruits inoculated with *C. siamense* and on day 2 in fruits inoculated with *C. asianum* (Figure 6). The pathogenicity and virulence of fungi can vary due to the genetic factors of the hosts and the environmental conditions in which they are found [2,6].

In a previous work reported by Jiménez-Maldonado et al. [2], the authors found that *C. siamense* was more virulent than *C. asianum* in Ataulfo mango fruits during postharvest, with mycelial growths of 33 and 28 mm, respectively, after 10 days of fruit storage at 28 °C. It was also reported that the highest peak of respiratory activity occurred at 2 dpi, which was accelerated by the presence of these two *Colletotrichum* spp. This correlates with *GLUC* and *CHIT* class IV expression from day 2 onwards, with *C. asianum* inoculation. In contrast, in mango fruit inoculated with *C. siamense*, their expression was triggered at 1 dpi because it was more virulent than *C. asianum*. An increase in ethylene and CO_2_ has been reported to coincide with fruit ripening and induces germination and the formation of the appressorium of *Colletotrichum* spp., but this only occurs in climacteric fruits [52,53,54]. The asymptomatic phase of anthracnose (a biotrophic fungal infection) occurs 48 h after inoculation, during which the species acquires nutrients from the infected host before necrosis; 72 to 96 h after the initial penetration of *Colletotrichum* spp., necrotrophic secondary hyphae develop that degrade the plant cell wall [55]. Therefore, the necrotrophic development of *Colletotrichum* spp. occurs at 3–4 dpi, which induces constant gene expression at 4 dpi in fruit subjected to both pathogens (Figure 6).

**Figure 7:**
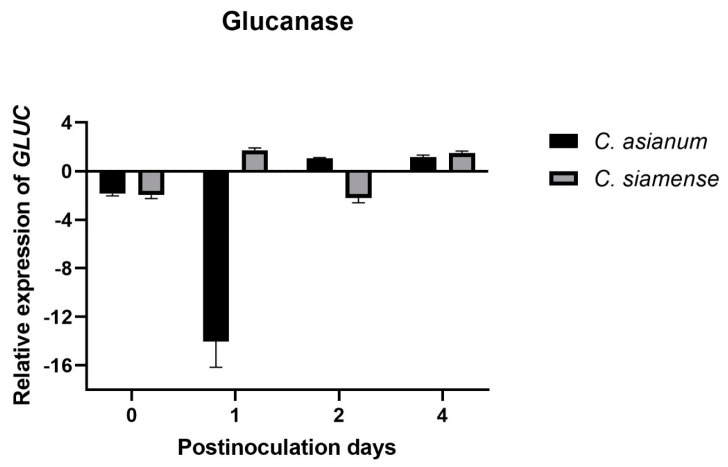

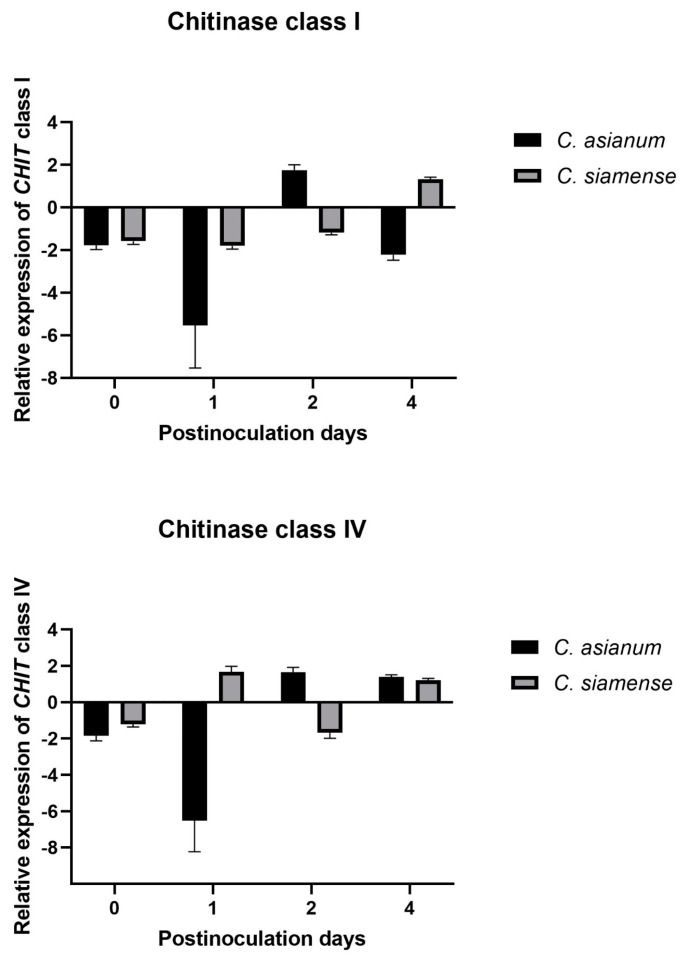
Expression of *GLUC* and *CHIT* class I and IV of *M. indica* induced by *C. asianum* and *C. siamense*. Uninoculated mango fruits were used as internal control. The days of analysis were 0, 1, 2, and 4 days post-inoculation (dpi) for RNA extraction and the assay by real-time quantitative reverse transcription polymerase chain reaction. Gene expression was normalized to the glyceraldehyde-3-phosphate dehydrogenase (GAPDH) gene [57] using the 2^−∆∆Ct^ method [58]. Data are shown as mean ± SD, analyzed by one-way ANOVA/Tukey test.

The primary hyphae of *Colletotrichum* spp., which have a hemibiotrophic mode of infection, are formed during its biotrophic phase. Subsequently, during its necrotrophic phase, secondary hyphae are formed [11]. Oliveira-Garcia and Deising [56] reported that β-glucan is necessary to stiffen the cell wall in fast-growing appressoria and the necrotrophic hyphae of *Colletotrichum graminicola* in maize. However, its synthesis is downregulated during biotrophic development. *Colletotrichum siamense* is more virulent and acts quickly to penetrate its host. It may be synthesizing β-glucans for the formation of an appressorium, explaining why the expression of glucanase in fruit subjected to *C. siamense* was positively induced at 1 dpi and again at 4 dpi (Figure 7). In contrast, the *C. asianum* isolate, which was less virulent, evaded the immunity caused by the downwardly synthesized β-glucan, and its *GLUC* expression was therefore induced at 2 dpi (Figure 7).

Furthermore, in rice, fungi such as *Magnaporthe oryzae*, *Cochliobolus miyabeanus*, and *Rhizoctonia solani* mask chitin and β-1,3-glucan (only in *M. oryzae*) in the fungal cell wall with α-1,3-glucan, preventing their enzymatic digestion by plant chitinases and glucanases and, thus, delaying the release of PAMPs [9,59]. α-1,3-glucan synthase genes are present in the genomic sequences of fungal pathogens, such as *C. graminicola*, *Myco-sphaerella graminicola*, *Puccinia graminis*, *Sclerotinia sclerotiorum*, and *Botrytis cinerea* [59]. This is also a strategy that *Colletotrichum* spp. may use to be detected early or late.

*CHIT* class IV was induced at the earliest dpi (1 and 2). However, *CHIT* class I was induced on the same day (2 dpi) when *CHIT* class IV was expressed in mango fruit inoculated with *C. asianum*; this is compared to the fruit inoculated with *C. siamense*, in which the expression of the *CHIT* class I gene was induced at 4 dpi (Figure 7). Some studies on rice plants provide information on the targets of chitinases, and these report that, in the early stage of pathogenesis, class IV chitinases release elicitor molecules from the fungal cell wall, and GlcNAc oligosaccharides activate the defense system. Subsequently, class I chitinases degrade the newly synthesized chitin chain of the fungus, inhibiting its growth [21,60]. The results in mango inoculated with *C. siamense* coincide with those reported in tomato inoculated with *F. oxysporum*, in which class IV chitinases were highly induced within 4 dpi and class I chitinases were positively induced only at 8 dpi [17]. We can also propose that *C. siamense* evades the mango immune system by delaying the expression of *CHIT* class I, causing larger necrotic spots to appear in the fruit [2].

Subnanomolar concentrations of GlcNAc oligosaccharides are sufficient to induce defense responses [61]. However, some studies have indicated that *Colletotrichum* spp. can convert exposed chitin to its nonacetylated chitosan derivative, preventing plant recognition of chitin fragments or PAMPs. This may occur in fruit inoculated with *C. siamense* compared to *C. asianum*, which is immediately recognized by class IV and I chitinases at 2 dpi [56,62,63]. Chitosan is a poor substrate for chitinase, and its fragments are less active as elicitors than chitin. Therefore, chitin deacetylation by *C. siamense* may interfere with its recognition by host chitinases [56,62,63]. 

Karunanayake et al. [64] reported that chitinases in latex and mango peel can rapidly degrade the conidial wall of *C. gloeosporioides*. An increase in the activity of chitinase and glucanase enzymes has also been reported from 2 to 8 dpi in mango fruits infected with anthracnose [23]. The genes that encode PRs have shown an expression on a basal level that increases drastically during a fungal infection. By expressing these genes, better adaptation to pathogen stress and greater plant performance have been observed [64,65]. Therefore, the overexpression of PRs genes, such as *GLUCs* and *CHITs*, improves plant resistance to diseases caused by fungi [24,42,66,67,68]. 

The results of this research can help develop effective control strategies to improve postharvest mango quality and avoid losses caused by these phytopathogens. We have provided important information about the classification of glucanases and chitinases to elucidate some of their functions in mango.

## 3. Materials and Methods

### 3.1. Classification of Glucanases and Chitinases from M. indica

#### 3.1.1. Identification of Glucanases and Chitinases

The genome (https://mangobase.org) of mango cv. Tommy Atkins from project PRJNA450143 [25] was blasted for sequences coding for glucanases and chitinases. The transcriptome of mango cv. Ataulfo (PRJNA286253) and orthologous sequences of *Arabidopsis* were used to obtain these gene families sequences from mango. 

The sequences of 23 glucanases and 16 chitinases were obtained from the genome of mango cv. Tommy Atkins [25] and used to search for their homologous and orthologous sequences in the NCBI database. Subsequently, PSI-BLAST (http://www.ncbi.nlm.nih.gov/BLAST, accessed on 4 January 2023) was performed to compare their amino acid sequences through alignments, and their regions of similarity were located using NCBI’s conserved domain (CD) search tool. Functional analyses of the proteins and predictions of conserved domains and sites were performed using the online EMBL-EBI (http://www.ebi.ac.uk/interpro/ (accessed on 4 January 2023)—European Molecular Biology Laboratory, 2023) and Scan Prosite programs (https://prosite.expasy.org/scanprosite/ (accessed on 4 January 2023)—ExPASy, 2023). We used the standardized *Arabidopsis* nomenclature of 25 GH9 family amino acid sequences for the glucanases [27] and 24 GH18 and GH19 family sequences for the chitinases [18,30]. As additional information, the names of the corresponding transcripts of mango cv Ataulfo (PRJNA286253) were mapped to the genomic proteins and are indicated in parentheses (Table 2 and Table 4).

#### 3.1.2. Sequence Alignment and Phylogenetic Analysis

Phylogenetic and molecular evolutionary analyses of mango cv. Tommy Atkins chitinases and glucanases were performed to classify them into GH families and classes or subclasses. Since the three families have different sequences and catalytic domains, phylogenetic analyses were performed separately for GH9, GH18, and GH19. MUSCLE and ClustalW were used for multiple alignments of the full lengths of the amino acid sequences [69,70], and MEGA-X 2021 was used to construct phylogenetic trees [50]. Bootstrap consensus trees for the heuristic search and their evolutionary history were inferred using the neighbor-joining (NJ) method [71], and evolutionary distances were calculated using the model based on the Jones–Taylor–Thornton (JTT) matrix with 1000 replicates start.

### 3.2. Glucanases and Chitinases from M. indica with Possible Defense Functions

The analysis for the selection of mango glucanases and chitinases induced by *C. siamense* and *C. asianum* was carried out using orthologous sequences reported in response to the defense against fungi. For glucanases, sequences that hydrolyze endo-β-1,3 bonds with a genomic background in *Arabidopsis thaliana*, *Ziziphus jujuba*, *Solanum tuberosum*, *Nicotiana tabacum*, *Triticum aestivum*, *Avena sativa*, *Oryza sativa*, and *Hordeum vulgare* were used. To select chitinases, sequences containing a chitin-binding domain (CBD) were searched in *A. thaliana*, *Morus notabilis*, *Pinus monticola*, *S. tuberosum*, *O. sativa*, and *H. vulgare*. Due to the classification of glucanases and chitinases, only the amino acid sequences of the glucanases Manin04g017870 and Manin20g000250 and class I chitinases Manin06g001290 and Manin09g013100 were used; of the class IV chitinases, we used Manin10g004580, Manin00g015200, Manin02g000400, Manin02g000410, and Manin05g002520. These glucanases and chitinases from mango cv. Tommy Atkins and their respective orthologous sequences were compared and aligned using MUSCLE and ClustalW with default settings [69,70]. The NJ method was used for the bootstrap consensus trees [71], and evolutionary distances were calculated using the JTT model, with a bootstrap value of 1000 repetitions in MEGA-X 2021 [50].

### 3.3. Prediction of cis-Acting Elements in Promoter Regions of the Glucanase and Chitinases Genes from M. indica

The CiiiDER tool [72] and the JASPAR 2022 open-access database of transcription factor binding profiles [73] were used to predict the *cis*-acting elements in the promoter regions (1500 bp upstream of the start codon) of the *GLUC* and the *CHIT* class I and IV of *M. indica* (selected from the genome of mango cv. Tommy Atkins).

### 3.4. Gene Expression Analysis of Glucanases and Chitinases in Mango cv. Ataulfo Infected with Anthracnose

#### 3.4.1. Vegetal Material

Mango cv. Ataulfo fruits, uniform in size and lacking visual damage, were selected from a commercial orchard in Los Mochis, Sinaloa, Mexico (DD 25.9300010, -109.0957430), based on their color, state number 2, to ensure equivalent physiological maturity. The fruits were disinfested with 1% sodium hypochlorite for 2 min, rinsed, and sprayed with 70% ethanol [4].

#### 3.4.2. Inoculation and Storage of Mango cv. Ataulfo Fruits

Isolates of *C. siamense* (UACH 334) and *C. asianum* (UACH 310) were provided by Tovar-Pedraza et al. [4]. The fungal colonies were incubated at 28 °C for 10 days on potato dextrose agar culture medium (PDA, BD Bioxon, CDMX, Mexico). Wounds were made on the surface of the epicarp of the mango cv. Ataulfo fruits, which were disinfested before, and mycelial discs of 5 mm diameter were emplaced [4,74]. A batch of mango cv. Ataulfo was inoculated with *C. siamense* and another with *C. asianum*, and control fruits without inoculation and without visible damage were included. All fruits were stored at 28 °C with a relative humidity of 85–90% for 4 days.

#### 3.4.3. RNA Extraction, cDNA Synthesis, and Relative Expression of Glucanases and Chitinases Genes

The expression levels of the genes involved in the defense mechanism in mango cv. Ataulfo were quantified by qRT-PCR. Two 5 cm diameter mango peel sections were cut for 3 biological replicates per treatment (2 inoculated and 1 noninoculated) at 0, 1, 2, and 4 dpi. Sections were frozen, sprayed with liquid nitrogen, and stored at −80 °C for analysis. Total RNA was extracted from mango epicarp (peel) tissue following the method described by López-Gómez and Gómez-Lim [75] with modifications from Dautt-Castro et al. [57]. RNA was treated with the Turbo DNA-free kit (Invitrogen, Carlsbad, CA, USA) to remove contaminating genomic DNA. cDNA was synthesized by reverse transcription using the SuperScript III First Strand kit (Invitrogen, CA, USA) following the manufacturer’s manual.

Quantitative PCR was carried out using the iTaq Universal SYBR Green Supermix kit (Bio-Rad, Hercules, CA, USA). All samples were amplified in triplicate PCR reactions that included 100 ng of template cDNA. Specific primers to amplify the glucanase and chitinases genes and the internal reference normalizing gene, glyceraldehyde-3-phosphate dehydrogenase (GAPDH) [57], are shown in Table 4. PCR products were amplified on a QIAxpert-QUIAGEN system. The conditions for amplification were 1 cycle of 95 °C for 10 min and 40 cycles of 95 °C for 15 s and 60 °C for 1 min. The specificity of the PCR product was confirmed by constructing a melting curve after amplification by raising the temperature to 60 °C for 1 min and then gradually to 95 °C for 15 s. Relative gene expression was calculated using the 2^−∆∆Ct^ [58].

#### 3.4.4. Statistical Analysis

For gene expression analysis, standard deviations were calculated from three biological replicates with three technical replicates. The results were analyzed using one-way analysis of variance (ANOVA) with a significance level of 5% and a Tukey test for differences in means; the NCSS 2024 version 24.0.2 statistical package was used.

## 4. Conclusions

Our study provides important information about the classification of glucanases and chitinases in *M. indica*. The genome sequence of mango cv. Tommy Atkins (*M. indica*) (PRJNA450143) identified 23 glucanases and 16 chitinases. In total, 19 glucanases were classified as β-1,4-glucanases of the GH9 family, while 4 could not be classified because they possessed different catalytic domains. Of the classified glucanases, five were subclass A, twelve were subclass B, and two were subclass C. A total of four chitinases were classified in the GH18 subfamily and ten in the GH19 subfamily. Overall, two class I, three class II, five class IV, and four class V chitinases were identified. Two β-1,3-1,4-glucanases and two class I and five class IV chitinases were identified in mango and were studied for their expression analysis in response to *Colletotrichum* spp.

We identified and demonstrated the different expression profile of the *GLUC* and *QUIT* class I and IV genes of mango cv. Ataulfo in response to infection induced by *C. siamense* and *C. asianum*, species of the *C. gloeosporioides* complex. Both the stimulation of plant’s innate immunity through the release of PAMPs from the fungal cell wall and the expression of plant glucanases and chitinases could potentially be used against diseases, such as anthracnose in mango, to create resistant cultivars through elicitors or genetic modifications.

## Figures and Tables

**Table 1:** Characterization of glucanases from *Mangifera indica*.

Sequence ID Genome * (Transcriptome) **	Description	Size (aa)	Glycosyl Hydrolase Domain	Catalytic Domain	Subclass	Cytosolic Domain	Transmembrane Domain	Signal Peptide	Carbohydrate Binding Module (CBM49)
Manin05g003870.1 (c35418_g1_i1)	Endoglucanase 16, hydrolase activity, carbohydrate metabolic process.	487	GH9	GH9: 34–478	A	NO	NO	1–28	NO
Manin09g009300.1 (c19892_g1_i1)	Endoglucanase 25, hydrolase activity, carbohydrate metabolic process.	621	GH9	GH9: 111–585	A	Cytosolic domain CT: 1–72	73–95	NO	NO
Manin15g000090.1 (c21105_g1_i1)	Endoglucanase 10, hydrolase activity, carbohydrate metabolic process.	524	GH9	GH9: 55–509	A	Cytosolic domain CT: 1–11	12–31	NO	NO
Manin15g008160.1 (c19892_g1_i1)	Endoglucanase 25, hydrolase activity, carbohydrate metabolic process.	608	GH9	GH9: 120–575	A	Cytosolic domain CT: 1–82	83–104	NO	NO
Manin18g012690.1 (c35418_g1_i1)	Endoglucanase 12, hydrolase activity, carbohydrate metabolic process.	891	GH9	GH9: 153–625, 633–690, 697–872	A	Cytosolic domain CT: 1–117	118–137	NO	NO
Manin04g000320.1 (c19965_g1_i2)	Endoglucanase, hydrolase activity, carbohydrate metabolic process.	686	GH9	GH9: 262–676	B1	NO	NO	NO	NO
Manin09g002870.1 (c10916_g1_i2)	Endoglucanase 8, cellulase activity, starch, and sucrose metabolic process.	499	GH9	GH9: 39–492	B1	NO	NO	1–30	NO
Manin11g004660.1 (c35418_g1_i1)	Endoglucanase 17, hydrolase activity, carbohydrate metabolic process.	476	GH9	GH9: 43–275, 280–466	B1	NO	NO	1–24	NO
Manin13g000270.1 (c19965_g1_i1)	Endoglucanase 24, hydrolase activity, carbohydrate metabolic process.	503	GH9	GH9: 37–493	B1	NO	NO	1–34	NO
Manin17g000910.1 (c10916_g1_i2)	Endoglucanase-like hydrolase activity, metabolic process of hydrolyzed carbohydrates of O-glycosyl compounds.	497	GH9	GH9: 35–481	B1	NO	NO	1–22	NO
Manin17g001950.1 (c19965_g1_i1)	Endoglucanase CX, hydrolase activity, carbohydrate metabolic process.	501	GH9	GH9: 35–489	B1	NO	NO	NO	NO
Manin20g006870.1 (c12199_g1_i1)	Endoglucanase 8, hydrolase activity, carbohydrate metabolic process.	922	GH9	GH9: 24–443, 471–915	B1	NO	NO	1–20	NO
Manin00g015190.1 (c12199_g1_i1)	Endoglucanase 4, hydrolase activity, carbohydrate metabolic process.	480	GH9	GH9: 24–471	B1	NO	NO	1–21	NO
Manin06g005470.1 (c12199_g1_i1)	Endoglucanase 2, hydrolase activity, carbohydrate metabolic process.	539	GH9	GH9: 68–526	B2	NO	NO	NO	NO
Manin11g009610.1 (c10916_g1_i1)	Endoglucanase 11, hydrolase activity, carbohydrate metabolic process.	525	GH9	GH9: 46–505	B2	NO	NO	1–43	NO
Manin12g007480.1 (c10916_g1_i1)	Endoglucanase 11, hydrolase activity, carbohydrate metabolic process.	539	GH9	GH9: 60–519	B2	NO	NO	1–36	NO
Manin14g009340.1 (c21105_g1_i1)	Endoglucanase 2, integral component of membrane cellulase activity, starch, and sucrose metabolic process.	426	GH9	GH9: 2–411	B2	NO	NO	NO	NO
Manin04g001090.1 (c35418_g1_i1)	Endoglucanase 6, hydrolase activity, carbohydrate metabolic process.	627	GH9	GH9: 30–489	C	NO	NO	1–26	535–615
Manin17g001380.1 (c35418_g1_i1)	Glucanase family 2, hydrolase activity, carbohydrate-binding carbohydrate metabolic process.	618	GH9	GH9: 29–488	C	NO	NO	1–25	526–606
Manin09g012550.1 (c22667_g2_i1)	Xylanase-like exoglucanase, hydrolase activity, carbohydrate metabolic process.	597	GH10	GH10: 47–595, 243–540 They hydrolyze glycosidic bonds between two or more carbohydrates	-	-	32–52	1–22	NO
Manin13g003320.1 (c22667_g2_i1)	Xylanase-like exoglucanase, hydrolase activity, carbohydrate metabolic process.	1095	GH10	GH10: 542–1071, They hydrolyze glycosidic bonds between two or more carbohydrates	-	-	-	Greater Facilitating Superfamily (MFS): 12–533	Galactose binding domain: 595–680
Manin04g017870.1 (c13218_g1_i1)	Endo-1,3-1,4-β-D-glucanase, hydrolase activity.	446	Hydrolase α/β	α/β hydrolase: 53–234, 235–444	-	NO	NO	NO	NO
Manin20g000250.1 (c24163_g1_i1)	Probable endo-1,3(4)-β-glucanase ARB_01444, endo-1,3-β-glucanase glucan activity, C-3 substituted reducing group.	894	GH81	Endo-β-glucanase: 60–704, 758–839 GH81: N: 89–363, 777–840; C: 369–704	-	NO	NO	1–59	NO

Mango cv. Tommy Atkins genome * (PRJNA450143) and mango cv. Ataulfo transcriptome ** (PRJNA286253). Not found: -.

**Table 2:** Characterization of chitinases from *Mangifera indica*.

Sequence ID Genome * (Transcriptome) **	Description	Size (aa)	Glycosyl Hydrolase Domain	Catalytic Domain	Class	Lysozyme Function	Signal Peptide	Chitin-Binding Domain (CBD) Region
Manin10g004580.1 (c18874_g2_i1)	PR4 endochitinase, cell wall macromolecule catabolic process.	718	GH19	GH19 with deletions	IV	YES	1–28	29–64, 296–331
Manin16g013090.1 (c26375_g1_i1)	Chitinase 10, cell wall macromolecule catabolic process.	265	GH19	GH19: 13–265	II	YES	1–23	NO
Manin00g015200.1 (c21654_g1_i1)	Chitinase IV, cell wall macromolecule catabolic process.	260	GH19	GH19 with deletions	IV	YES	1–28	29–64
Manin02g000400.1 (c21682_g1_i1)	Endochitinase EP3, response to wounds, response to bacteria, hypersensitive response, somatic embryogenesis, cell wall macromolecule catabolic process.	934	GH19	GH19 with deletions	IV	YES	1–26	31–50, 86–105, 316–335, 369–388, 618–637, 657–676, 700–719
Manin02g000410.1 (c21682_g1_i1)	Endochitinase EP3, cell wall macromolecule catabolic process.	466	GH19	GH19 with deletions	IV	YES	1–23	23–58, 265–300
Manin02g001370.1 (c16890_g1_i1)	Chitinase 1 response to water stress, salt stress response, lignin biosynthetic process, cell growth, response to metabolic nitrate regulation of salicylic acid.	320	GH19	GH19: 67–293	II	YES	1–24	NO
Manin02g010580.1 (c16890_g1_i1)	Chitinase 2, cell wall macromolecule catabolic process.	321	GH19	GH19: 69–296	II	YES	1–23	NO
Manin05g002520.1 (c21654_g1_i1)	Chitinase tipo 1, cell wall macromolecule catabolic process.	485	GH19	GH19 with deletions	IV	YES	1–23	236–271
Manin06g001290.1 (c25021_g3_i3)	Pentatricopeptide chitinase containing At2g17670 repeats, cell wall macromolecule catabolic process.	720	GH19	GH19: 89–191, 200–210	I	YES	1–23	24–65
Manin09g013100.1 (c26375_g1_i1)	Like endochitinase, cell wall macromolecule catabolic process.	310	GH19	GH19: 70–295, 208–218	I	YES	1–20	21–62
Manin05g000480.1 (c37799_g1_i1)	Chitinase 3, 1, protein kinase activity, ATP binding, protein phosphorylation.	563	GH18	GH18: 66–412 Protein kinase: 288–563, Ser/Thr kinase: 462–474	V	NO	-	NO
Manin16g014070.1 (c24038_g1_i1)	Chitinase isoform X2, cell wall macromolecule catabolic process.	869	GH18	Chitinase 2: 104–424, GH18: 104–440	V	YES	1–36, 37–58	NO
Manin07g009130.1 (c22170_g1_i1)	Chitinase 3, 1.	129	GH18	GH18: 1–105	V	NO	-	NO
Manin07g009180.1 (c22170_g1_i4)	Like mammalian acid chitinase, cell wall biogenesis.	892	GH18	Chitinase 2: 544–875, GH18: 544–892	V	NO	1–20	NO
Manin15g007800.1 (No transcript)	Like endochitinase A.	306	-	-	-	-	-	-
Manin07g009150.1 (c23148_g1_i2)	Acidic isoform like mammalian chitinase X2.	160	-	-	-	-	1–34	-

Mango cv. Tommy Atkins genome * (PRJNA450143) and mango cv. Ataulfo transcriptome ** (PRJNA286253). Not found: -.

**Table 3:** Fungal-induced glucanases and chitinases associated with plant defense.

Plant	Glucanase (β-1,3-Glucanase)	Inducing Pathogen	References
Jujube	AAY25165.1	*Cryptococcus laurentii*	Tian et al., [43]
*A. thaliana*	AAM67102.1	77% ID with jujube	* Tian et al. [43]
	NP_001325845.1 (At3g57260)	*Botrytis cinérea*, *Erysiphe cichoracearum*, *Erysiphe orontii*	Doxey et al. [44]
	NP_191283.2 (At3g57240)	*Erysiphe cichoracearum*, *Erysiphe orontii*	Doxey et al. [44]
Potato	pir||S31196	49% ID with jujube	Tian et al. [43] *
Wheat	AAY96422.1	*Rhizoctonia* sp.	Liu et al. [45]
	CAA77085.1	*Fusarium graminearum*, *Alternaria* sp., *A. glaucus*, *A. flavus*, *A. niger*, *Penicillium* sp.	Zhang et al. [46]
	AAD28734.1	*Bipolaris sorokiniana*	Aggarwal et al. [47]
Oatmeal	AAP33176.1	80% ID with wheat	* Liu et al. [45]
Rice	AAL35900.1	74% ID with wheat	* Liu et al. [45]
Barley	AAM75342.1	94% ID with wheat	* Liu et al. [45]
Tobacco	BAB17320.1	41% ID with jujube	* Tian et al. [43]
Corn	NP001148461.1 (β-1,3-1,4-glucanase)	---------	Perrot et al. [20]
**Plant**	**Chitinase** **(Classes I y IV)**	**Inducing Pathogen**	**References**
Blackberry	EXB44469.1 class I EXB55191.1 class IV EXB55192.1 class IV	*Botrytis cinerea*	Xin et al. [40]
*A. thaliana*	NP_566426.2 class I AAP88360.1 class IV	*Botrytis cinerea*	Xin et al. [40]
	AAA32769.1 class I		Sasaki et al. [21]
	NP_191010.1 class IV		Liu et al. [48]
White Pine	AAS83984.1 class IV	*Cronartium ribicola*, rust fungus	Liu et al. [48]
Tea (potato gene overexpression)	AAF25602.1 class I	*Phytophthora infestans* (blight) in *Camellia sinensis* (Tea)	Singh et al. [49]
			Kumar et al. [50]
Litchi (rice gene overexpression)	CAA38249.1 class I	*Phomopsis* sp. in litchi	Das et al. [42]
Carrot (barley gene overexpression)	AAA18586.1 class I	*Alternaria radicicola* and *Botrytis cinerea* in carrot	Jayaraj and Punja, [41]

% ID: identity percentage. Jujube (* Tian et al. [43]) and wheat (* Liu et al. [45]) share significant identity with β-1,3-glucanase identified in plants like *A. thaliana*, potato, tobacco, oats, rice, and barley, suggesting a possible defense response against fungi such as *Cryptococcus laurentii* and *Rhizoctonia* sp.

**Table 4:** Primers were used for relative expression analysis of mango glucanases and chitinases.

	Primers
Manin20g000250.1 ß-1,3-1,4-Glucanasa	F: 5′CAGGATTTCACCCGAGAGAATAG3′
	R: 5′TCAGGAGGAGCAAACCAAAG3′
Manin05g002520.1 Quitinasa class IV	F: 5′GCTCCCAACTTGTGTTGCAG3′
	R: 5′CCCCTTACACCCCAATCCAC3′
Manin09g013100.1 Quitinasa class I	F: 5′CCTCCAAGAGCTTCTACAGTTAC3′
	R: 5′CATGGGAAGTTTGGGCTAAGA3′

F: forward, R: reverse.

## Data Availability

Data are contained within the article.
